# Free-Roaming Dog Population Estimation and Status of the Dog Population Management and Rabies Control Program in Dhaka City, Bangladesh

**DOI:** 10.1371/journal.pntd.0003784

**Published:** 2015-05-15

**Authors:** Tenzin Tenzin, Rubaiya Ahmed, Nitish C. Debnath, Garba Ahmed, Mat Yamage

**Affiliations:** 1 Food and Agriculture Organization of the United Nations, Emergency Centre for Transboundary Animal Diseases, Dhaka, Bangladesh; 2 Disease Prevention and Control Unit, National Centre for Animal Health, Thimphu, Bhutan; 3 Obhoyaronno-Bangladesh Animal Welfare Foundation, Mohammadpur, Dhaka, Bangladesh; The Global Alliance for Rabies Control, UNITED STATES

## Abstract

Beginning January 2012, a humane method of dog population management using a Catch-Neuter-Vaccinate-Release (CNVR) program was implemented in Dhaka City, Bangladesh as part of the national rabies control program. To enable this program, the size and distribution of the free-roaming dog population needed to be estimated. We present the results of a dog population survey and a pilot assessment of the CNVR program coverage in Dhaka City. Free-roaming dog population surveys were undertaken in 18 wards of Dhaka City on consecutive days using mark-resight methods. Data was analyzed using Lincoln-Petersen index-Chapman correction methods. The CNVR program was assessed over the two years (2012–2013) whilst the coverage of the CNVR program was assessed by estimating the proportion of dogs that were ear-notched (processed dogs) via dog population surveys. The free-roaming dog population was estimated to be 1,242 (95 % CI: 1205–1278) in the 18 sampled wards and 18,585 dogs in Dhaka City (52 dogs/km^2^) with an estimated human-to-free-roaming dog ratio of 828:1. During the two year CNVR program, a total of 6,665 dogs (3,357 male and 3,308 female) were neutered and vaccinated against rabies in 29 of the 92 city wards. A pilot population survey indicated a mean CNVR coverage of 60.6% (range 19.2–79.3%) with only eight wards achieving > 70% coverage. Given that the coverage in many neighborhoods was below the WHO-recommended threshold level of 70% for rabies eradications and since the CNVR program takes considerable time to implement throughout the entire Dhaka City area, a mass dog vaccination program in the non-CNVR coverage area is recommended to create herd immunity. The findings from this study are expected to guide dog population management and the rabies control program in Dhaka City and elsewhere in Bangladesh.

## Author Summary

Rabies is a public health problem in Bangladesh. A CNVR program was commenced in Dhaka City in January 2012 as part of the Bangladesh national rabies control program. We describe the findings of a dog population survey that was conducted to estimate the free-roaming dog population using a mark-resight framework and the progress of the CNVR program. The free-roaming dog population in the 18 sampled wards was estimated to be 1,242 dogs, and 18,585 dogs in Dhaka City (52 dogs/km^2^). The estimated human-to-free-roaming dog ratio was 828:1. Between 2012 and 2013, 6,665 dogs (3,357 male, 3,308 female) were neutered and vaccinated against rabies in 29 of the 92 city wards. A pilot survey conducted in 18 wards indicated a CNVR coverage of 60.6% (range 19.2–79.5%). We recommend conducting an annual mass dog vaccination in the non-CNVR coverage area during the intervening period of the CNVR program to create herd immunity against rabies and break the cycle of rabies transmission.

## Introduction

Rabies kills an estimated 2,000–2,500 people every year in Bangladesh, ranking it third globally after India and China in terms of human impact [1–4]. In Bangladesh an estimated 166,590 (95% CI: 163,350–170,550) cases of animal bites in humans are reported each year, contributing to an estimated annual incidence of 1.40 human rabies deaths per 100,000 population [3]. The Infectious Disease Hospital (IDH) located in Dhaka City is the main referral centre for rabies patients in Bangladesh; it provides free treatment to 350 to 450 dog bite victims daily [1, 5]. For example, between 2004 and 2012 the IDH in Dhaka City reported 1,152 human rabies deaths [1, 5]. At the district level, 65 rabies prevention and control centers provided free anti-rabies vaccine and treatment to dog bite victims [5]. In domestic animal populations, 3,425 rabies deaths (cattle: 2845; goats: 547; sheep: 13) were reported in a passive surveillance system (2010–2012) in Bangladesh [6]. However, rabies cases in dogs were not captured by this surveillance system and other reliable data are scarce. The mortality in both animals and humans may be several fold higher than reported since rabies is not a notifiable disease in Bangladesh [1].

In Bangladesh, domestic dogs act as the main source of rabies for both domestic animals and humans [1,3]. Until late 2011, mass dog culling was implemented in major towns in Bangladesh, in an unsuccessful attempt to control rabies [1]. For example between 2003 and 2008 there were 139,391 stray dogs culled in five major towns (Dhaka, Khulna, Rajshahi, Sylet and Tongi) in Bangladesh, of which 80% (n = 112,078) were culled in Dhaka City alone (average 22,415 dogs per year) [1], yet rabies infection remained endemic. Therefore, Obhoyaronno-Bangladesh Animal Welfare Foundation (OBAWF) carried out an advocacy campaign against mass culling of dogs and recommended a humane method of dog population and rabies control in Dhaka City. Subsequently, the government approved and provided support and commitment to the OBAWF to implement a long-term humane dog population management and rabies control program in Dhaka City through Catch-Neuter-Vaccinate-Release (CNVR). Since January 2012, mass culling of dogs was stopped in Dhaka City. On 1 April 2012, OBAWF signed a memorandum of understanding with both Dhaka North and South City Corporations to assume the responsibility of managing Dhaka City’s entire dog population humanely. OBAWF carried out CNVR as part of the National Rabies Control program in Dhaka City.

Knowledge of the size of the dog population is crucial for dog management and for assessing the effectiveness of dog population and rabies control strategies. However, no studies have been conducted to estimate the size of the free-roaming dog population in Dhaka City. In addition, since the start of the CNVR program in January 2012, no assessment has been made of the coverage of the CNVR program in Dhaka City.

The objectives of this study were to: (1) estimate the size of the free-roaming dog population using a mark-resight framework, (2) describe the status of CNVR program conducted during 2012–2013, and (3) estimate the proportion of free-roaming dogs that had been sterilized and vaccinated against rabies in the CNVR program in Dhaka City. The findings from this study are expected to guide the dog population management and rabies control program in Dhaka City and elsewhere in Bangladesh.

## Materials and Methods

### Study area

**Fig 1:**
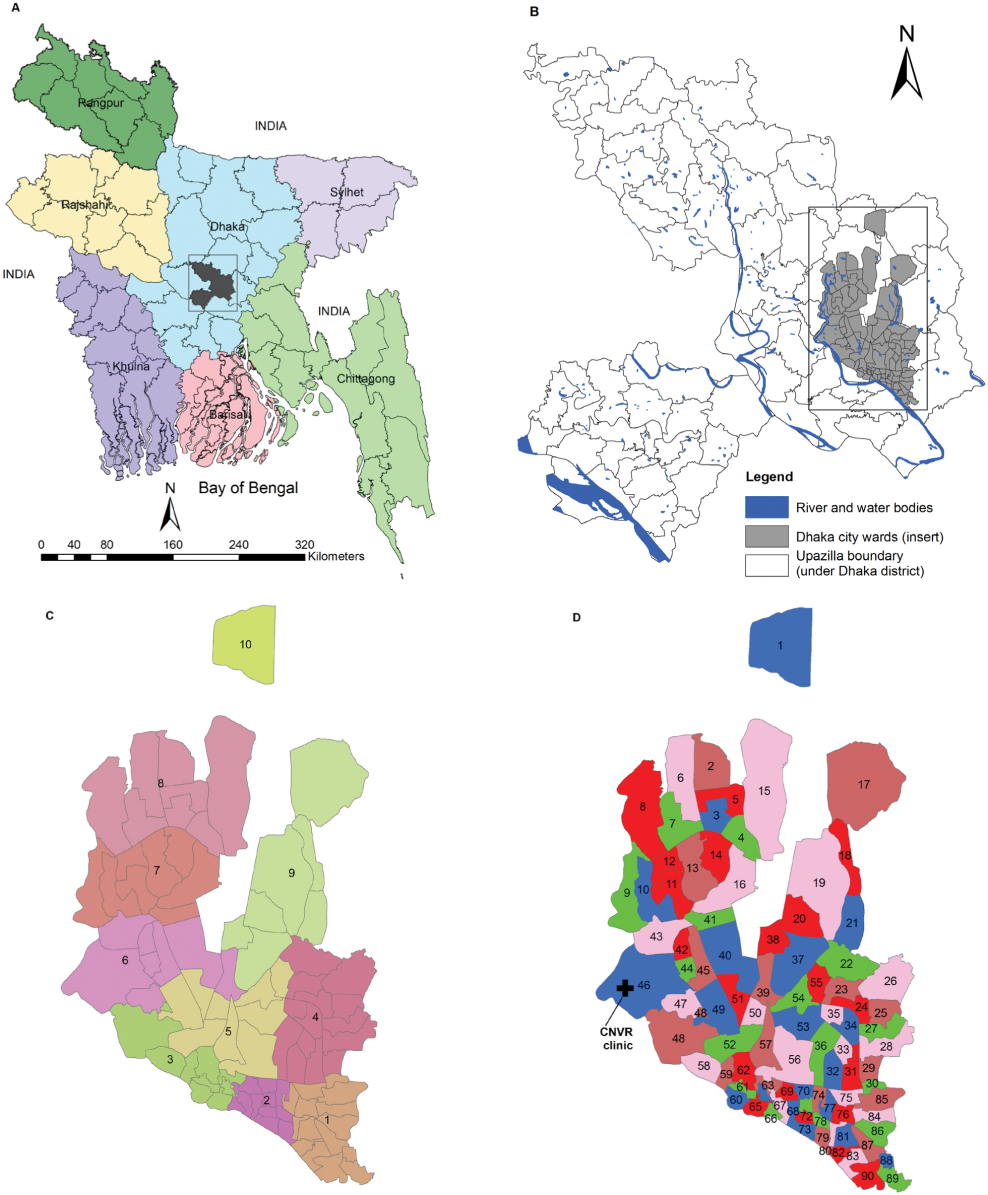
Panel A: Map of Bangladesh showing the 7 Divisions (Rangpur, Rajshahi, Dhaka, Sylhet, Chittagong, Khulna, Barisal), 64 districts boundary and Dhaka district (shaded part within Dhaka division); Panel B: Dhaka district showing different Upazilla (sub-district) boundary with river and water bodies and Dhaka City wards (shaded); Panel C: Dhaka City divided into 10 zones (Zone 1 to 5: Dhaka South City Corporations and Zone 6 to 10: Dhaka North City Corporation); Panel D: Dhaka City wards with respective ward number and free-roaming dog population surveys have been conducted in red colour wards (see Materials and Methods). CNVR program were conducted in 29 wards during 2012–2013 and is ongoing to cover the entire wards. CNVR clinic is located in ward No 46.

The People's Republic of Bangladesh is located in South Asia. It is divided into seven administrative divisions (Barisal, Chittagong, Dhaka, Khulna, Rajshahi, Sylhet, Rangpur) and 64 districts. Each district is further divided into Upazilla (sub-district) while the metropolitan areas are divided into wards (Fig 1, panel A-D).

Dhaka is the capital of Bangladesh and is also the principal city of Dhaka Division and Dhaka District. The city is administered by the Dhaka City Corporation (DCC) which is divided into two administrative parts—North and South Dhaka City Corporations—ensuring better civic facilities. The city is divided into 10 zones and further sub-divided into 92 wards (Fig 1, panel C and D). An estimated 15.391 million people live in Dhaka City and it is one of the most densely populated cities in the world [7].

### Free-roaming dog population estimation

In this paper, free-roaming dogs are defined as any dogs seen in public areas that are not confined to its owner's house or property and are not currently under direct human control. Therefore, this term encompasses owned, unowned and community owned dogs but not for those on leashes or under direct human control at the time of survey [8]. However, the proportion of owned dogs that are typically free-roaming is unknown in Dhaka City, but is likely that some owned dogs are free-roaming.

During January to March 2011, a mark-re-sight procedure was used to estimate the free-roaming dog population in Dhaka City. As part of the sampling process, a polygon map for each ward was coloured with one of five colors: brown, red, green, pink and blue according to World Society for the Protection of Animals (WSPA) guidelines (Fig 1, panel D) [8]. A lottery was then used to decide which of the denoted *wards* would be chosen for surveys. Red colour polygons were selected and these consisted of 19 wards. Then a detailed route map of each of the 19 selected wards were prepared for the survey based on the neighbourhood road networks. Some of the larger wards were split and sub-divided into smaller, more manageable blocks for the survey.

Three two-person survey teams were formed for the population survey. Each team was assigned wards and fixed routes to follow on a daily basis for the survey. On day 1, the teams travelled by motorcycle through their predetermined routes and sprayed water-soluble blue colour vegetable dye on to the dogs observed in the street, without capturing the dogs. A farmers' sprayer machine was used for marking the dogs. The harmless colour mark remains on the dog body for at least two weeks and can be easily resighted from a distance during secondary sampling. The team made three to five visits to mark the dogs within the designated neighbourhood. At the time of marking, the team recorded the total number of dogs marked in each ward. The number of dogs marked with colour on the first day represented the ***n***_***1***_ within the mark-resight framework [9]. On the second day (7.00–12.00 h), each team returned to the same location/neighbourhoods where the dogs were marked on the previous day and carried out counting of free-roaming dogs. All survey teams walked through the street in one direction without overlapping the area to avoid double counting of dogs. During the counting process, the teams recorded the presence of a paint mark on the body of sighted dogs. The total number of dogs counted on day 2 forms the ***n***_***2***_ while the number of colour marked dogs resighted forms ***m*** in the mark-resight framework (Table 1). The same process of marking and counting event was done in all wards sequentially.

**Table 1:** Estimation of the number of free-roaming dogs and dog density in Dhaka City, Bangladesh during January and March 2011.

Ward No	Area (sq.km)	n_1_	m	n_2_	N (95% CI)	Dog density (dogs/sq.km)
5	1.336	74	48	77	118 (107–130)	88
8	3.937	66	47	69	97 (89–105)	25
11	1.572	115	42	71	193 (164–222)	123
12	1.614	117	80	121	177 (164–189)	110
14	2.859	92	71	102	132 (124–140)	46
18	1.466	15	5	14	39 (21–57)	27
20	2.388	56	36	55	85 (76–95)	36
24	2.157	47	34	45	62 (57–67)	29
31	1.922	6	4	8	12 (8–15)	6
38	1.523	104	71	94	138 (129–146)	91
42	1.096	22	11	26	51 (36–65)	47
55	0.023	20	13	17	26 (22–30)	1115
62	0.401	27	21	25	32 (30–35)	80
65	0.404	2	1	2	4 (2–5)	10
69	0.109	10	7	9	13 (11–15)	120
72	0.427	3	2	2	3 (3–4)	7
76	0.624	22	14	18	28 (24–32)	45
82	0.021	18	11	20	32 (25–39)	1544
Total	23.880	816	518	775	1242* (1205–1278)	52

*n*_*1*_: total number of animals sighted and marked on the first sample

*n*_*2*_: total number of animals sighted on the second sample

*m*: number of marked animals on the first sample that were re-sighted on the second sample

*N* is the estimated total population of dogs using Chapman estimates with 95% confidence interval (see Eqs 1 and 2 in the text).

*Total estimated population is the sum of the estimate in each ward

The Lincoln—Petersen’s formula with Chapman’s correction was applied to estimate the free-roaming dog population [10].

$$N=\left[\frac{\left(n_{1}+1)(n_{2}+1\right)}{\left(m+1\right)}\right]-1$$ (1)

where, ***N*** is the estimate of the total population size, ***n***_***1***_ is the total number of animals sighted and marked on day 1, ***n***_***2***_ is the total number of animals sighted on day 2, and ***m*** is the number of marked animals on day 1 that were sighted on day 2.

An approximately unbiased variance of *N* was estimated by using Seber's formula [9]:

$$\mathrm{var}(N_{})=\left[\frac{\left(n_{1}+1)(n_{2}+1)(n_{1}-m)(n_{2}-m\right)}{{\left(m+1\right)}^{2}(m+2)}\right]$$ (2)

And the 95% confidence interval for *N* was estimated as:

$$N\pm 1.965\sqrt{\mathrm{var}(N)}$$ (3)

The 18 surveyed wards and DCCs had an approximate area of 23.880 and 126.59 km^2^ respectively. The total number of free-roaming dogs within the areas of DCC was estimated by adjusting the total estimated free-roaming dog population in the studied wards with ward area sampling fraction (1242 ÷ 23.880/126.59 = 1242/0.1886) [7]. To estimate the free-roaming dogs in the Dhaka metropolitan area (DMA), a total density of dogs estimated per km^2^ in the surveyed wards was adjusted with the entire metropolitan area (357.42 km^2^) by assuming homogeneity of the area in terms of habitat and food resource availability for free-roaming dogs within the city. The analysis was conducted using Microsoft Excel (Microsoft Excel 2007, Redmond, USA).

### CNVR program

The first CNVR program in Dhaka City commenced in January 2012 and was implemented by Obhoyarono-Bangladesh Animal Welfare Foundation. The CNVR program was conducted in 18 wards (Ward No 7 to 14; 39, 40, 43 to 50) during 2012 and in 29 wards (Ward No 2 to 14; 39 to 50; 54, 55, 58, 60) during 2013 of Dhaka North City Corporation (Table 2). The CNVR program is ongoing and will be continued until all 92 wards in Dhaka City have been covered, based on the memorandum of understanding signed between OBAWF and DCCs.

**Table 2:** Total number of dogs processed at a capture-neuter-vaccinate-release (CNVR) clinic between January 2012 and December 2013 and estimated sterilization and vaccination coverage in 18 wards in Dhaka City, Bangladesh (January-February 2014).

	CNVR program during 2012–2013					Pilot assessment of CNVR coverage			
Ward No	Male	Female	Total (%) ^a^	Year of CNVR	N^b^	Un-notched ^c^	Notched ^d^	Total counts ^e^	CNVR coverage (95% CI) ^f^
2	32	18	50 (0.75)	2013	3	161	68	229	29.69 (24.15–35.91)
3	50	26	76 (1.14)	2012; 2013	5	126	30	156	19.23 (13.81–26.12)
4	46	20	66 (0.99)	2013	5	279	111	390	28.46 (24.21–33.13)
5	88	54	142 (2.13)	2013	8	134	106	240	44.17 (38.02–50.49)
6	78	101	179 (2.69)	2013	10	250	130	380	34.21 (29.62–39.12)
8	115	91	206 (3.09)	2012; 2013	13	125	152	277	54.87 (48.99–60.63)
9	192	227	419 (6.29)	2012; 2013	28	94	319	413	77.24 (72.95–81.02)
13	96	44	140 (2.10)	2012; 2013	9	125	106	231	45.89 (39.58–52.33)
14	167	87	254 (3.81)	2012; 2013	15	84	222	306	72.55 (67.29–77.25)
41	87	106	193 (2.90)	2012; 2013	11	77	169	246	68.70 (62.65–74.17)
43	282	328	610 (9.15)	2012; 2013	45	83	318	401	79.30 (75.07–82.99)
46	488	553	1041 (15.62)	2012; 2013	83	275	915	1190	76.89 (74.41–79.20)
47	139	82	221 (3.32)	2012; 2013	14	53	147	200	73.50 (66.98–79.13)
48	655	658	1313 (19.70)	2012; 2013	84	149	422	571	73.91 (70.15–77.34)
50	22	14	36 (0.54)	2012	3	171	43	214	20.09 (15.27–25.97)
54	49	55	104 (1.56)	2013	5	15	4	19	21.05 (08.50–43.33)
55	26	35	61 (0.92)	2013	3	183	429	612	70.10 (66.35–73.59)
58	61	112	173 (2.60)	2013	10	113	153	266	57.52 (51.51–63.31)
7	108	54	162 (2.43)	2012; 2013	10				
10	130	209	339 (5.09)	2012; 2013	19				
11	185	152	337 (5.06)	2012; 2013	23				
12	55	77	132 (1.98)	2012; 2013	7				
39	24	22	46 (0.69)	2012; 2013	3				
40	18	28	46 (0.69)	2012; 2013	3				
42	11	9	20 (0.30)	2013	1				
44	5	6	11 (0.17)	2012	1				
45	110	104	214 (3.21)	2012; 2013	20				
49	31	26	57 (0.86)	2012; 2013	5				
60	7	10	17 (0.26)	2013	1				
Total	3357	3308	6665		447	2497	3844	6341	60.62 (59.41–61.81)

**Note:**

^a^ Total number of dogs processed with percent of total dogs processed in each ward.

^b^ N means number of visit (times) made in each ward during CNVR program to catch dogs and present to the clinic (e.g. in ward no. 2, the dog catching team visited 3 times on different days to catch dogs and present these to the CNVR clinic).

^c^ dogs that were not ear-notched (meaning not sterilized/vaccinated) and sighted by the counting team during field survey.

^d^ permanent V-shaped identification mark applied on ear of processed dogs under anesthesia during the CNVR program that were sighted by the counting team during field survey.

^e^ Total number of notched and un-notched dogs sighted and counted during field survey.

^f^ CNVR coverage is calculated as the total number of notched dogs sighted divided by total dog counts with 95% confidence interval. Only 18 wards were surveyed during January–February 2014. Ward No 55 population survey data includes dog counts from another ward with unknown number (this ward number has not been recorded during the pilot study).

Prior to the start of the program, four veterinary surgeons and four para-veterinarians were trained for two months (September–October 2011) by the HSI in India on a range of dog population management aspects including humane dog catching, aseptic surgical sterilization methods (ovario-hysterectomy and castration), monitoring of post operative complications, dog population survey and data management.

During the program, free-roaming dogs sighted in the street were humanely captured by the trained dog catchers either by hand or using nets in the morning (6.30–10.00 h) and brought using a mobile van to the clinic set up at Boshila, Mohamadpur (Ward no 46), under Dhaka North City Corporation (Fig 1, panel D). However, pregnant and lactating dogs were not captured for sterilization and vaccination. In few instances, the program had to be suspended whenever there was political unrest and strikes in the city, public holidays and also due to logistic constraints including shortage of drugs.

Dogs were classified as either owned, community or ownerless dogs. Owned dogs were brought in to the clinic by the owners; ownerless dogs were captured in the street by the dog catchers. When communities claimed that the dogs lived within their community and that they fed the dogs, they were classified as community dogs. All dogs were handled and neutered—including anesthetic and drug administration—according to the standard protocols developed by HSI. Each dog was given a rabies vaccine (Rabisin, Merial, France) injection. The dogs were also given an ivermectin (Techno Drug Ltd, Narsingdi, Bangladesh) injection to control internal and external parasites. A V-shaped ear notch was performed on all neutered and vaccinated dogs that were classified as ownerless or community dogs while under anesthesia to permanently identify processed dogs. The surgical procedure was completed before 14.00 h to ensure that all operated dogs were fully recovered from anesthesia before being released back to the place of capture in the same afternoon. Recovery from anaesthesia typically takes about two to three hours post operation. Any dog that displayed discomfort, weakness after surgery or took longer to recover from anesthesia were retained at the clinic and treated before being released back to their territory.

Two years’ (January 2012 to December 2013) data on the CNVR program were retrieved from the database and descriptive analyses were performed to understand the sex, ownership, and space-time pattern of dogs caught and processed in the clinic.

### A pilot assessment of CNVR coverage

During January and February 2014 a pilot assessment of CNVR coverage—via dog count surveys—was carried out in 18 of the 29 randomly selected wards of Dhaka North City Corporation (Ward No: 2, 3, 4, 5, 6, 8, 9, 13, 14, 41, 43, 46, 47, 48, 50, 54, 55 and 58) where the CNVR program was implemented during 2012–2013 (Table 2). Three teams each comprising of three persons walked predetermined routes through each selected ward and counted the number of dogs by direct observation (without capturing or handling dogs). For each dog counted, the teams recorded the details based on visual assessment: sex (male, female), presence of ear notch (yes, no), body (1—very thin, 2—thin, 3—normal, 4—stout, 5—overweight) and skin (normal—no observable skin lesions, mildly diseased—few skin lesions, moderately diseased—moderate number of skin lesions, severely diseased—severe skin lesion with apparent mange infestation) condition scores and the approximate age (pup—up to 6 months, juvenile—6 to 12 months and adults—more than 12 months) (Table 3). Counting was conducted between 6.30–12.00 h when dogs were most active and likely to be visible to the counting team.

**Table 3:** Health condition (body condition and skin condition score) of free-roaming dogs in Dhaka City, Bangladesh (January-February 2014).

Variables	Body condition scores counts (percent)						Skin condition score counts (percent)				
	1	2	3	4	5	Total	Normal	Mild	Moderate	Severe	Total
***Neuter status***											
Neutered	322 (8.54)	839 (22.25)	2269 (60.19)	319 (8.46)	21 (0.56)	3,770	2634 (71.73)	658 (17.92)	327 (8.91)	53 (1.44)	3,672
Intact	148 (6.05)	487 (19.89)	1543 (63.03)	256 (10.46)	14 (0.57)	2,448	1620 (67.16)	524 (21.72)	221 (9.16)	47 (1.95)	2,412
***Sex***											
Male	265 (7.68)	728 (21.10)	2158 (62.63)	273 (7.91)	27 (0.78)	3,451	2412 (71.19)	625 (18.45)	299 (8.83)	52 (1.53)	3,388
Female	198 (7.29)	583 (21.47)	1630 (60.04)	297 (10.94)	7 (0.26)	2,715	1813 (68.39)	543 (20.48)	247 (9.32)	48 (1.81)	2,651
***Age categories***											
Adult	356 (7.45)	990 (20.72)	3031 (63.45)	374 (7.83)	26 (0.54)	4,777	3391 (72.4)	835 (17.83)	380 (8.11)	78 (2.00)	4,684
Juvenile	28 (7.00)	77 (19.25)	252 (63.00)	38 (9.50)	5 (1.25)	400	242 (60.80)	107 (26.88)	42 (10.55)	7 (2.00)	398
Puppies	86 (9.28)	243 (26.21)	451 (48.65)	146 (15.75)	1 (0.11)	927	548 (61.30)	220 (24.61)	113 (12.64)	13 (1.00)	894

**Note:** body condition score scale: 1-very thin; 2-thin; 3-normal/ideal; 4-stout; 5-overweight. Skin condition score: Normal: normal skin without any observable skin lesion; mild: few skin lesions; moderate: moderate number of skin lesions on the body and severe: severe skin lesion with supposedly mange infestation/scabies.

Age categories: Up to 6 months: pup; 6 to 12 months: Juvenile and more than 12 months: adults.

Vaccination and sterilization coverage of the free-roaming dog population in the 18 sampled wards were estimated as the proportion of ear-notched dogs sighted and counted during the population survey. Descriptive analyses were performed to describe the male-to-female ratio and the health condition of the dogs.

## Results

### Free-roaming dog population estimation

Only 18 out of the 19 selected wards in Dhaka City could be surveyed: ward 90 could not be surveyed due to logistic constraints. A total of 816 dogs (*n*_*1*_) were marked with colour paint spray between January and March 2011 and 775 dogs (*n*_*2*_) were counted at the secondary count event; of these 775 dogs, 518 (*m*) were re-sighted with colour paint marks (Table 1). The re-sighting probability was 63%. Using the Lincoln-Petersen index-Chapman correction methods, the free-roaming dog population was estimated to be 1,242 (95% CI: 1205–1278) in the 18 sampled wards and 6,584 dogs within the DCC administrative areas (Table 1). The DMA area has an area of approximately 357.42 km^2^ and by using the dog density estimates from the sampled wards, the overall free-roaming dog population in DMA during early 2011 was estimated to be 18,585 dogs (52 dogs per km^2^). Using the human population of Dhaka City to be 15.391 million, the human-to-dog ratio was estimated to be 828:1.

### CNVR program

**Fig 2:**
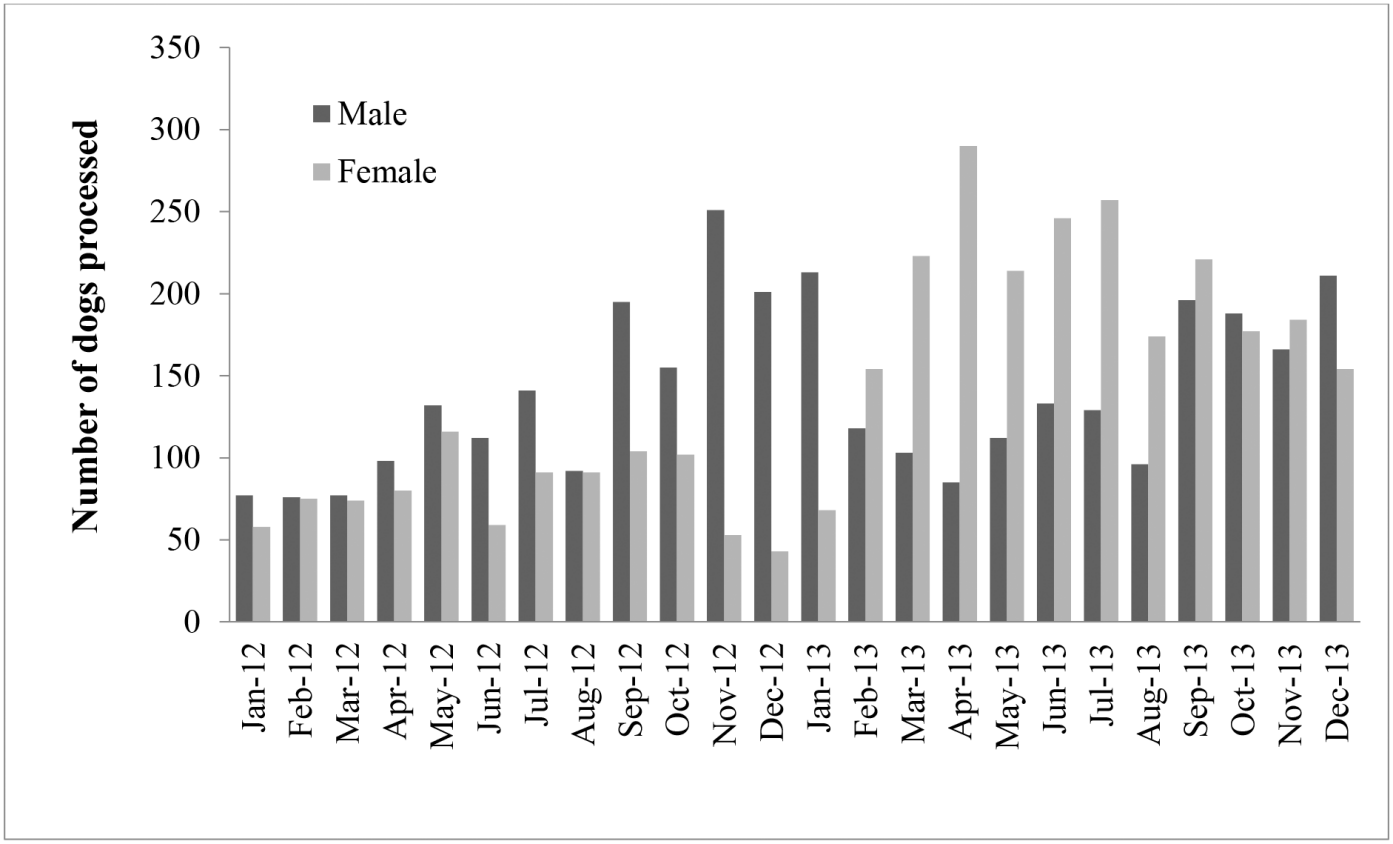
Monthly process and release of dogs after rabies vaccination and sterilization between January 2012 and December 2013 in Dhaka City, Bangladesh.

A total of 6,713 (2,587 in 2012 and 4,126 in 2013) dogs were caught and presented to the CNVR clinic for vaccination and sterilization between January 2012 and December 2013. Of these, 6,665 (99.3%) dogs (2553 in 2012 and 4112 in 2013) were processed (vaccinated, sterilized and released). The remaining 48 dogs had various health conditions and were thus unfit for surgical intervention, but nonetheless were given an anti-rabies vaccine injection. Almost equal number of male (3,357; 50.4%) and female (3,308; 49.6%) dogs were processed during 2012 and 2013 (male-to-female ratio 1.01:1). The majority of the dogs processed were classified as ownerless (5,266; 78%) whereas 14% (942) were owned and 8% (505) were community dogs. Fig 2 shows the gender-specific monthly pattern of dogs processed at the clinic during the two year period. The highest number of dogs (n = 2,354) were processed in wards 46 and 48 (Table 2). The relationship between frequency of visits and CNVR coverage in each ward is shown in Table 2.

### A pilot assessment of CNVR coverage

The survey team counted 6,341 dogs, of which 3,844 were found to be ear-notched, in 18 wards. The overall point estimate of vaccination and sterilization coverage was estimated to be 60.6% (95% CI: 59.4–61.8). Of the 18 wards, eight had an estimated coverage >70% (Table 2). There was a higher proportion of male (3,528) than female (2,759) dogs in the sampled wards (overall male-to-female ratio of 1.28:1). The CNVR coverage was 62.1% (2,191) in male and 58.9% (1,618) in female dogs.

The age distribution of ear-notched dogs was found to be skewed towards the adult groups (4,832 [78.3%]; 95% CI: 77.2–79.3), with fewer puppies (938 [15.1%]; 95% CI: 14.2–15.9) and juveniles (413 [6.6%]; 95% CI: 6.1–7.3). The estimated CNVR coverage in adults, puppies and juveniles was 63.4% (3,088), 50.7% (476) and 45.3% (187), respectively.

The majority of the free-roaming dogs sighted in this study had good body condition (score 3) and normal skin condition. Neutered dogs and male dogs had higher BCS and better skin condition that intact dogs and female dogs respectively (Table 3).

## Discussion

As far as we are aware, this was the first study conducted to estimate the size of the free-roaming dog population in Dhaka city, Bangladesh, using mark-re-sight methods. This information was needed to design the subsequent CNVR program, for example to estimate the doses of rabies vaccine and other drugs required and to determine how many dogs would need to be sterilized and vaccinated to control the dog population and to reduce the rabies transmission risk. However, this population survey was conducted in 2011 after a citywide mass dog culling operation conducted by the city corporations in which more than 22,400 dogs were eliminated every year (2003–2008) in an attempt to control rabies and reduce the dog population in Dhaka City [1]. The current study generated a point estimate of the dog population based on data collection during early 2011. It may therefore underestimate the existing population within the city because the mass dog culling campaign was replaced by a long-term sustainable dog population management and rabies control via CNVR in January 2012. Nevertheless, this baseline information can be used to understand the dog population size in Dhaka City and to compare future estimates.

The mark-resight method is considered to be a practical way of estimating the number and distribution of a free-roaming dog population, if the assumption of a closed population (no appreciable births, deaths, immigration and emigration of dogs) is fulfilled during the primary and secondary sampling intervals [9,11]. In this study, marking and subsequent counting events were completed within three days and thus the assumption of a closed population was likely valid because of the very short period between counting events. Only a few published studies have used mark-resight and sight-resight methods (by observing the natural body marks on dogs, counting permanently identifying features such as ear-notch status, collaring of dog or colour paint spray) to estimate the size of free-roaming dog populations [12–18]. The method chosen for population estimation will depend on the availability of resources and their practicality in the field. In the current study, we applied vegetable colour paint marks to dogs followed by secondary counting events and resight of the marked and unmarked dogs to estimate the free-roaming dog population. We have found previously that such colour paint marks remain on the dog's body for at least two weeks (even when it rains) and that the marked dogs can be easily sighted from a distance during the secondary resighting sampling process [17]. This method is quick and cheap and can be used for population surveys or to mark dogs during vaccination campaigns to assess the vaccination coverage when other methods are unavailable or impractical.

Within the DMA the estimated free-roaming dog population was 18,585 dogs and 52 dogs/km^2^, respectively. There are huge differences in estimated dog densities between wards (Table 1) which may be associated with variation in human density and the availability of food resources. The population of free-roaming dogs in Dhaka City cannot be assumed to be homogenous, an important finding for planning CNVR program and other disease response plans. Therefore, available resources could be used more effectively by focusing dog and rabies control measures in areas known to have higher dog density to achieve high vaccination coverage. The estimated human-to-free-roaming dog ratio of 828:1 is relatively high compared to international studies presumably due to high human density in Dhaka City. The dog density (dogs/km^2^) estimate in Dhaka City is moderate compared to other Asian cities which range from 5.78 in Timor Leste to 2,930 in Kathmandu, Nepal, with human-to-dog ratio ranging from 4.7:1 in Kathmandu to 23:1 in Timor Leste [15, 18, 19]. In contrast, Hossain et al., [4] estimated 14 dogs per km^2^ with human-to-dog ratio of 120:1 in rural Bangladesh. The density of dogs is expected to be lower in rural than urban areas. Also, estimates of the density of dogs may vary between different areas and countries due to socio-cultural differences and the type of dogs included in counts. In the current study, only free-roaming dogs (but not owned restrained dogs) were counted, although it is expected that some proportion of owned dogs might have been included in the survey.

During the two years (January 2012–December 2013) CNVR program in Dhaka City, more than 6000 dogs were processed with variable coverage between the neighbourhoods (Table 2). The teams, however, attempted to improve the coverage by making repeated visits to areas that had previously been targeted. For instance, during 2013 the CNVR program was re-focused on those neighbourhoods/wards which were already targeted during 2012 to increase the overall level of coverage (Table 2). However, some wards were visited only once (for example ward 42 and 60) during 2012 and 2013. This is because when the dog catching team assessed few free-roaming dogs within the neighborhood, the team moved to the next wards for capturing and in this way, the free-roaming dogs within the city wards were captured and taken to the clinic for processing. Nevertheless, there has been a consistent increase in the total number of dogs processed at the CNVR clinic from 2,553 dogs in 2012 to 4,112 in 2013.

Most dogs processed at the clinic were classified to be ownerless, rather than owned or community dogs. It is possible that owned dogs may not have been presented to the clinic for sterilization because the dog owners did not want their dogs to be sterilized, or the clinic was too far away and the owners did not have enough time to take their dogs. In some cases, owned dogs may have been presented to the government veterinary clinics or private clinics, although the service was provided free of charge at the CNVR clinic.

The point estimate for CNVR coverage among the free-roaming dogs in the sampled 18 wards was 60.6%. However, assuming that some owned dogs are vaccinated and neutered but not ear-notched and are free-roaming in the street, this CNVR coverage estimates may have been underestimated. Of these 18 wards, eight had an estimated coverage level >70%, exceeding the WHO recommended threshold for rabies eradication [20, 21]. Within these eight wards there were repeated visits by the dog catching teams and longer program duration. For example, ward numbers 43, 46 and 48 were visited 45, 83 and 84 times, respectively, by the dog catching teams and thus achieved higher coverage compared to other wards. Since city wards are demarcated mainly for administrative purposes and have no physical boundary, a certain amount of movement of dogs between the wards/neighborhoods is likely to have occurred during, as well as following, the CNVR program. For instance, a higher number of ear-notched dogs were observed in certain wards during the post-CNVR program assessment survey, compared to the initial number of dogs processed in those wards (Table 2), indicating movement of dogs between neighbourhoods.

Although almost equal number of male and female dogs were processed during CNVR program in 2012 and 2013, a higher number of male than female free-roaming dogs were found during a pilot assessment surveys conducted during early 2014. This may be due to higher survival of males than females as BCS of males were found to be better than females during a pilot study. The finding of more male than female dogs in this pilot assessment study was consistent with what has been reported elsewhere in the world where male dogs predominate [13, 17, 22, 23]. The information on sex-ratio of free-roaming dogs will be useful for planning the logistical arrangements for CNVR program in Dhaka City.

This pilot assessment study has helped to identify those wards/neighbourhoods during 2014 where the program coverage was low. Most of those wards with lower coverage and new wards have been covered during 2014. Such evaluation surveys are necessary to assess the coverage and then modify the program accordingly. In this survey, not all 29 wards could be included due to logistical reasons, which is why these results pertain to only 18 wards. Although there is no rule-of-thumb regarding what proportion of the dog population needs to be neutered to control and stabilize the dog population, WSPA recommend 70% coverage (similar to the target vaccination coverage for rabies elimination), but this will require long-term planning and sustained resources [13, 24]. Nevertheless, OBAWF have committed to cover the entire Dhaka City to manage the dog population. Since the CNVR program may take few more years to cover the entire Dhaka City dog population, mass dog vaccination against rabies is recommended in the non CNVR areas during the intervening period to create herd immunity and break the transmission chain of rabies virus. The CNVR program needs to cover more areas (city wards) and dogs within a short time period. This can be strategically achieved by setting up additional CNVR clinics in different wards to improve the level of coverage. This will be more efficient since collecting dogs from far off neighborhoods and transporting them to and from the existing clinic located at ward No 46 (Fig 1, panel D) takes considerable time and resources.

The majority of the free-roaming dogs sighted in the street had good BCS, with neutered dogs appearing healthier than intact dogs, and also males appearing healthier than female dogs (Table 3). The improvement of BCS and skin condition of the processed dogs could be due to the direct benefit of the CNVR program [25]. For instance, all dogs presented to the clinic were given one dose of ivermectin injection to control internal and external parasites and also treated against skin infections and other health ailments encountered during the processing. One of the objectives of the CNVR program is to improve the health and welfare of free-roaming dogs. The health benefits of sterilization in both males and females include preventing multiple pregnancies, reducing sexually transmissible diseases (such as transmissible venereal tumor), improving body weight and condition, and increasing life span [25–27]. In females repeated pregnancies can physically stress animals, while the absence of pregnancy can improve health and lifespan [25]. The most important benefit of sterilization is stabilization of the population by reducing the population turnover rate, but this requires a sustained program over a longer period of time. Because of a longer life span of neutered [25,26] and vaccinated dog, herd immunity against rabies is also maintained and not lost as rapidly between rounds of vaccination. It is expected that the health and welfare of free-roaming dogs may improve in the future as more dogs are covered by the CNVR program.

### Conclusions

This study provides baseline information about the dog population size and the status of CNVR coverage in Dhaka City. We recommend that indicator counts are conducted every year to assess the dynamics (size and distribution) of the dog population and CNVR coverage. Also, recording the number of dog bites in humans and incidences of rabies in the city would provide information about the impact of the program. Because the CNVR program will take time to cover the entire Dhaka city area simultaneously, annual mass dog vaccination is recommended in the non-CNVR coverage area to create herd immunity among the dog population and to break the transmission chain of rabies virus. In addition, the setting up of additional CNVR clinics (e.g mobile clinics) could increase coverage and reduce the dog population and rabies incidence, as well as improve the health and welfare of dogs in Dhaka City. There is also a need for inter-agency collaborations and the One Health approach engaging human health and veterinary professionals to control rabies in Bangladesh.
